# Rose Water

**Published:** 1908-01

**Authors:** 


### ROSE WATER.

The absurdity of certain features of the Georgia prohibition law has been recently revived by the construction placed upon the question of druggists selling certain preparations containing alcohol, but not suitable or possible for beverages.

A special effort was made by the physicians, last summer when the legislature was considering the passage of the present law, to have it more carefully worded so as not to impose a hardship upon druggists and citizens—temperate citizens who perhaps might desire some of the numerous everyday household remedies containing alcohol only as a preservative. All of the suggestions that the bill be more correctly drawn up so as to embody at least the views of its supporters, were met by the statement that it was too late to make any changes, as such an effort might defeat the bill entirely. Dr. Hardman admitted before the Fulton County Medical Society that it was not his intention to make the law so sweeping and that he hoped it would not be so construed. The fact, however, that the wording did not eliminate certain remedies that were never used as beverages, rendered the recent decision of Recorder Broyles and City Attorney Mayson that no concoction containing alcohol could be sold—except on the prescription of a physician—without breaking the law, was the natural outcome of the haste and unscientific spirit of the prohibitionists.

City Attorney Mayson calmly replied to the plea of the druggists that they "should have gone before the legislature and had this law drawn up otherwise," just as though every effort had not been made by the druggists and physicians to make the bill a reasonable one before passing it. Many of the physicians who believe in temperance, and some of them in prohibition, were forced, by their efforts to remodel this measure, to the position of being altogether opposed to prohibitory laws..

The present construction placed upon the law is the logical outcome of its literal reading; that anyone should have assumed that it could consistently be construed otherwise, when the language is so clear, seems preposterous.

The physicians—even the prohibition physicians, will be very glad to make careful examinations of the zealous temperance supporters, and, if deemed necessary, write a prescription for a small amount of rose water—perhaps more, if the condition seems to justify it—provided such prohibitionists do not think a fee of two dollars excessive.

